# Two Time Point Analysis of the Change in Risk and Aging Factors for Major Cancers: A 10-Year Longitudinal Study in China

**DOI:** 10.1155/2020/9043012

**Published:** 2020-05-06

**Authors:** Liu Hui

**Affiliations:** Department of Clinical Immunology, Dalian Medical University, Dalian 116044, China

## Abstract

**Objective:**

To quantify the change in risk and aging factors with a two time point analysis for major cancers to assess supportive strategies.

**Methods:**

The 2004 and 2015 mortality statistics in China were accessed. The standardized mortality rates of the two periods were used to calculate the ratio of change (RC) value to assess the risk of death associated with time (social development with time) for cancers. The role of age in mortality with time was evaluated by the interaction between time and age using a Poisson regression.

**Results:**

In ascending order of RC, the factors were uterus; other malignant neoplasms; esophagus; stomach; skin; liver; leukemia; “lip, oral cavity, and pharynx”; bladder; “colon and rectum”; breast; prostate; lung; ovary; pancreas; “lymphoid, hematopoietic, and related tissue”; and cervix cancers. According to their location on the scatter diagram, the 17 neoplasms could be divided into three groups, comprising undeveloped cancers (including four cancers), developed cancers (including three cancers), and cancers insensitive to social development. Unexpectedly, about 60% (as assessed by type of cancer) and two-thirds (as assessed by constituent ratio of death from all cancers) of cancers did not change with time.

**Conclusions:**

Most cancers may be insensitive to social development. Internal factors, including aging, may be a key factor for the occurrence of cancer.

## 1. Introduction

Cancers are complex disorders, and it is virtually impossible to prove what causes a particular cancer, because most cancers have multiple possible causes. However, we can classify cancer-related factors as risk or protective factors to assess preventative strategies. A hitherto unknown number of risk and protective factors contribute to cancer development by interacting with each other. Although cancer can develop at any age, cancer incidence increases with age [1–3]; therefore, it is believed that old age is the most important risk factor for cancer development and was treated as a major variable in the present study.

Worldwide, the rates of cancer are increasing, mainly related to the aging population and changes in lifestyle resulting from social development [4–6]. Some cancers seem to be more prevalent in people with a lower quality of life, whereas other cancers may be more prevalent in people with a higher quality of life [7]. Social development or urbanization will result in changes to cancer risk and protective factors, including aging, diet and obesity, lack of physical activity, smoking, radiation, infections, stress, and environmental pollutants [8–10], and their impact on the occurrence of cancer [11–13]. Therefore, in growing economies, whether the time trend of increased social development might influence the development and burden of cancer should be explored, which will allow us to identify and implement appropriate medical strategies. To date, there have been no comprehensive and quantitative two time point analyses on cancers.

**Figure 1:**
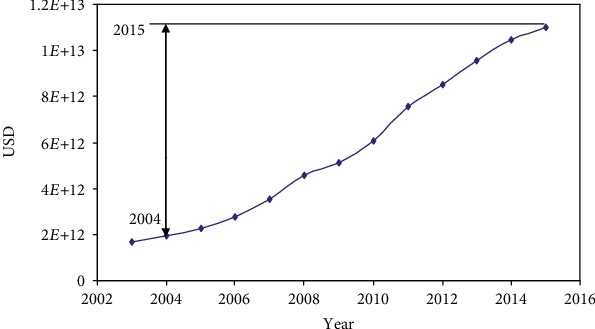
Gross domestic product from 2003 to 2015 in China.

The cancer incidence (201.7/100000) in China is similar to that of the overall worldwide incidence (197.9/100000) in 2018 [14]. China's ethnic composition is relatively simple and is presently undergoing rapid urbanization [15–17]. China's gross domestic product was 1.955 trillion USD in 2004 and 11.016 trillion USD in 2015 (Figure 1). Health and medical care has been markedly affected by economic growth, making China an ideal model to study disease patterns associated with modernization [18–20]. In this study, we analyzed the characteristics of cancers using two properties: (1) age [8] and (2) risk factors [7]. The results showed that although these two properties varied significantly among some cancers, the rates of most cancers were unchanged with time. We also categorized 17 major cancers using two time point analysis and found that about 30% of cancers were sensitive to social development, and more than 60% of cancers were insensitive to social development. An improved understanding of such associations would benefit the development of a theoretical basis for cancer prevention and control strategies.

## 2. Materials and Methods

### 2.1. Original Data

Original data was obtained from the 2004 and 2015 data sets of the national disease mortality surveillance system, which was edited by the Chinese Center for Disease Control and Prevention [21, 22], representing data obtained from over 73 million out of the 1.4 billion population of China between 2004 and 2015. In 2004, life expectancy at birth was 73.6 years, whereas in 2015, it was 76.1 years. The International Classification of Diseases- (ICD-) 10 codes [23] were used to classify the underlying causes of death, allowing us to determine the mortality statistics. Tables 1 and 2 show the raw data.

### 2.2. Two Time Point Analysis of Deaths Caused by Neoplasms

The mortality rates in 2004 and 2015 were standardized using the 2000 Standard in China [7] and were used to calculate the ratio of change (RC) with time with respect to deaths caused by a particular cancer. The RC values were calculated using the following formula:

(1) $$\begin{array}{c} RC=\frac{P_{1}}{P_{0}}, \end{array}$$

where *P*_1_ represents a cancer's mortality rate in 2015 and *P*_0_ represents the cancer's mortality rate in 2004.

An RC value > 1 indicated that the mortality rate had increased with time, whereas a value < 1 indicated a decreasing trend with time. To evaluate the degree of deviation from 1 (the values greater than 1 and those less than 1 were comparable), logarithmic transformation was used to transform the RC values into logarithms, with the absolute value, lnRC, serving as the quantitative value for the two time point analysis.

A scatter diagram was plotted using the lnRC values. The degree of deviation from 1 and the positions of the various cancers on the scatter diagram were used to sort neoplasms according to the changes in the mortality rate with time.

### 2.3. Assessing the Role of Age on Mortality from Cancer over Time

To evaluate the mortality risk from aging and social development, a loglinear model using the Poisson regression was employed, with covariates of time (2004 and 2015) and a categorical age of 60 years old [7]. Poisson regression is a generalized linear model form of regression analysis used to model count data and contingency tables. Poisson regression assumes the response variable *Y* has a Poisson distribution and assumes the logarithm of its expected value can be modeled by a linear combination of unknown parameters. From the model, the *B* coefficients for the interaction between time and age were obtained, in which exp (*B*) was taken as the risk ratio. The greater the positive value of *B*, the greater the effect of the aging factor with time was on a particular cancer [7].

A probability of a type I error ≤ 0.05 was considered statistically significant. SPSS ver. 17.0 software for Windows (IBM, Armonk, NY, USA) was used to analyze the data statistically.

## 3. Results

The two time point analysis of deaths caused by neoplasms is summarized and quantified in Table 3. The contributions of time (social development) to different cancers varied: The lnRC value for the 17 neoplasms ranged from −1.04 to 0.38. The greater the lnRC, the greater the disease risk over time. In ascending order of lnRC were uterus; other malignant neoplasms; esophagus; stomach; skin; liver; leukemia; “lip, oral cavity, and pharynx”; bladder; “colon and rectum”; breast; prostate; lung; ovary; pancreas; “lymphoid, hematopoietic, and related tissue”; and cervix cancers.

Table 4 shows the results of the Poisson regression analysis to evaluate the mortality risk of aging with time (social development) for major cancers. We observed variations in the quantitative values of the effect of age with time on diseases. For the 17 cancers, the *B* value (the interaction between time and age) ranged from −0.169 to 0.435. A higher value of *B* indicated a stronger effect on cancer of aging with time (social development). The effect of aging with time was significantly reduced for cancers of the uterus, liver, pancreas, and cervix (*p* < 0.05) and was significantly increased for cancers of the esophagus and leukemia (*p* < 0.05).

**Figure 2:**
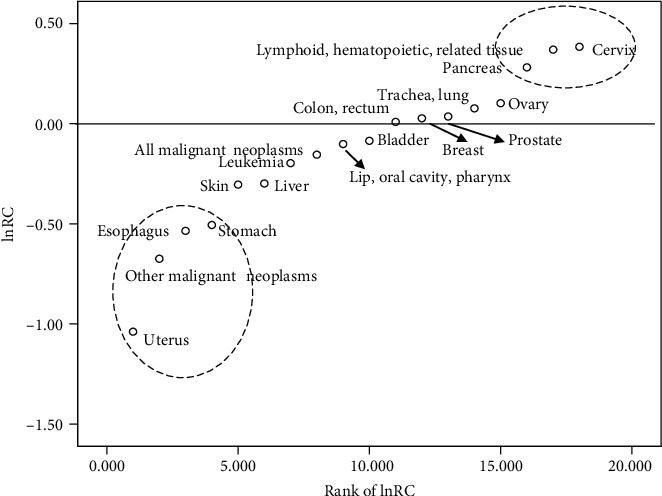
Cancer groups classified on the basis of sensitivity to time. RC: ratio of change with time; rank of lnRC: in order of cancers with lnRC with respect to deaths caused by a particular cancer.

According to their positions on the scatter diagram, the 17 neoplasms could be suggested to divide into three groups (Figure 1). Cancers located in the upper right were termed developed cancers (more prevalent in people with a higher quality of life) and comprised three cancers. Cancers in the lower left part were termed undeveloped cancers (more prevalent in people with a lower quality of life) and comprised four cancers. The third group, positioned between the developed and undeveloped groups, was termed as cancers insensitive to social development and comprised ten cancers (58.8%).  Figure 2 illustrates the detailed changes with social development for the three groups of cancers.

**Figure 3:**
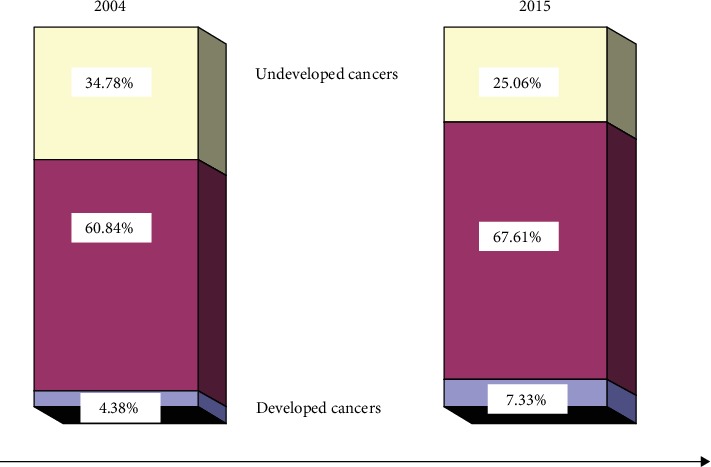
Changes in the constituent ratio with time for the three groups of cancer.

The constituent ratios of death from the three groups of cancers are shown in Figure 3. The constituent ratio increased from 4.9% in 2004 to 7.33% in 2015 for the developed cancers, decreased from 34.78% in 2004 to 25.06% in 2015 for the undeveloped cancers, and was approximately equal (60.84% in 2004 vs. 67.61% in 2015) for cancers insensitive to social development. The constituent ratios of death from insensitive to social development cancers were approximately two-thirds of all cancers.

## 4. Discussion

Life expectancy at birth in 2004 was 73.6 years, and in 2015, it was 76.1 years. Therefore, our data for the two time point analysis could reveal the contribution of social development to the development of cancer. The results revealed that these contributions varied without consideration of age. In the 17 cancers studied, the lnRC value ranged from −1.04 to 0.38 (Table 3), indicating that the contributions of social development to different cancers varied.

The greater the lnRC, the greater the risk of disease risk with time (social development). The lnRC data showed that among the 17 cancers studied, uterus cancer had the lowest disease risk with time (social development) and cervix cancer had the highest risk. The 17 neoplasms could be divided into developed cancers (for which social development might be a risk factor), undeveloped cancers (for which lack of social development might be a risk factor), and cancers insensitive to social development, according to their position on the scatter diagram (Figure 2). The constituent ratio of death from all cancers increased from 4.9% in 2004 to 7.33% in 2015 for developed cancers and decreased from 34.78% in 2004 to 25.06% in 2015 for undeveloped cancers (Figure 3). This suggested that the categories of cancer in this study are accurate and reasonable.

Unexpectedly, about 60% (as assessed by type of cancer) and two-thirds (as assessed by constituent ratio of death from all cancers) of cancers were insensitive to social development, which suggested that the improvement in social development and medical care might have played a limited role in altering the rate of death from cancers. Internal factors, including aging, might have a key effect on cancer occurrence.

Aging is a significant and uncontrollable risk factor for death [1, 24, 25]; therefore, determining the effects of aging on cancers with social development is important. The different contributions of age to mortality from cancers between 2004 and 2015 could also be considered as the change in the effect of other risk factors on cancers with time (social development). A larger difference indicated an increased effect of aging or a decreased effect of other risk factors on death resulting from cancers with social development. Therefore, changes in the effect of aging with time implied that the structure of the risk factors had changed with increasing social development. Our results showed that the *B* value (representing the interaction between time and age) for the 17 cancers ranged from −0.169 to 0.435. The effect of aging with social development was significantly reduced for cancers of the uterus, liver, pancreas, and cervix (*p* < 0.05) and was significantly increased for cancers of the esophagus and leukemia (*p* < 0.05), suggesting that the structure of risk factors had changed with increasing social development for these cancers. Moreover, for most of the cancers categorized as insensitive to social development, the level of risk and the structure of the risk factors had not changed with time (Figure 3).

The identification of a group of developed cancers, including cervix, pancreas, and “lymphoid, hematopoietic, and related tissue” cancers, showed that risk factors from social development have contributed to increases in the rates of some cancers. The risk factors might have a more important role in the relatively young population for cervix and pancreas cancers. Accordingly, an emphasis on avoiding risk factors from social development or urbanization might contribute to disease prevention for these developed cancers.

The presence of a group of undeveloped cancers, including uterus, esophagus, stomach, and other malignant neoplasms, showed that protective factors from social development have contributed to decreases in the rates of some cancers. The protective factors might have a more important role in the relatively young population for esophagus cancer and in the relatively old population for uterus cancer. Accordingly, disease prevention might be aided by an emphasis on public health and increasing basal health status for undeveloped cancers.

One limitation of this study is that the social development was not described with more indicators. Time and social development may not be equal. However, China's gross domestic product was 1.955 trillion USD in 2004 and 11.016 trillion USD in 2015; in 2004, life expectancy at birth was 73.6 years, whereas in 2015, it was 76.1 years. Therefore, time and social development may be parallel during 2004-2015. Increase of gross domestic product and rapid urbanization would change lifestyle; cancer-related risk would also be changed with this processes. Therefore, time could represent social development in this period. The findings also suggested that only about 30% of cancers are sensitive to changes in socioenvironmental factors over time. Analyzing and controlling external risk factors might lead to 30% of cancers being prevented, because the external factors leading to cancers are relatively controllable. Solutions to developed urbanized diseases mainly involve avoiding risk factors, which include tobacco use, overweight, physical inactivity, inadequate diet, sexually transmitted infections, alcohol consumption, and air pollution. General protective interventions for life quality, including nutritional status, lifestyle, and quality of social life, could potentially delay the aging process. Thus, the primary prevention of cancers will be aided by increasing our understanding of the categories of cancer affected by social development. Further studies are warranted to observe correlation between specific risk factors with social development and cancers for developing more available preventative strategies.

## Figures and Tables

**Table 1:** Age-stratified number of deaths from major cancers in 2004 in China [21].

Age	Cancers																		Survival
	A	B	C	D	E	F	G	H	I	J	K	L	M	N	O	P	Q	R	
0-	0	0	0	0	1	0	0	2	0	0	0	0	0	0	2	35	13	53	688115
1-	1	0	0	0	7	0	0	2	0	0	0	0	0	1	3	69	6	89	3030920
5-	0	0	0	1	7	0	2	2	0	0	0	0	0	0	5	95	5	117	4604931
10-	1	0	0	3	16	0	2	0	0	2	0	2	1	1	9	146	9	192	6443682
15-	10	0	7	12	34	2	22	2	1	1	2	5	1	0	16	220	13	348	5900624
20-	11	8	15	24	64	0	18	2	5	2	5	1	4	1	15	157	14	346	5467254
25-	24	11	53	47	141	6	60	3	20	6	14	9	1	1	19	125	24	564	6299615
30-	69	27	123	83	422	20	165	11	55	32	35	12	2	5	22	163	35	1281	6992522
35-	114	81	302	123	777	41	365	12	136	65	69	13	0	9	54	205	44	2410	6360862
40-	149	189	436	148	1144	45	575	13	205	104	104	32	4	18	52	149	67	3434	5173403
45-	232	471	867	282	1810	96	1034	22	254	102	135	59	8	24	71	203	78	5748	5115691
50-	337	852	1320	374	2190	141	1709	25	339	108	231	80	14	31	131	211	101	8194	4047509
55-	305	1102	1761	430	2193	166	1950	30	238	117	166	50	19	41	110	154	126	8958	2957812
60-	304	1427	2166	509	2120	241	2523	38	168	94	156	51	26	82	121	169	99	10294	2486122
65-	359	1784	2816	686	2430	276	3363	60	184	102	201	62	74	132	150	192	150	13021	2125649
70-	320	2046	3175	876	2232	328	3961	69	136	83	153	50	145	185	157	178	147	14241	1601637
75-	183	1568	2526	751	1692	269	3045	68	127	64	120	38	140	186	123	126	128	11154	1022733
80-	137	967	1571	525	1011	154	1755	65	72	59	83	25	124	149	80	77	109	6963	545697
>85	72	472	817	332	527	67	930	57	42	35	36	18	104	112	30	39	65	3755	308427

Neoplasms (ICD-10 codes): A: lip, oral cavity, pharynx (C00–C14); B: esophagus (C15); C: stomach (C16); D: colon, rectum (C18–C21); E: liver (C22); F: pancreas (C25); G: trachea, lung (C33–C34); H: skin (C43–C44); I: breast (C50); J: cervix (C53); K: uterus (C54–C55); L: ovary (C56); M: prostate (C61); N: bladder (C67); O: lymphoid, hematopoietic, related tissue (C81–C90, C96); P: leukemia (C91–C95); Q: other malignant neoplasms (C17, C23–C24, C26–C32, C37–C41, C45–C49, C51–C52, C57–C60, C62–C66, C68–C80, and C97); R: all cancers.

**Table 2:** Age-stratified number of deaths from major cancers in 2015 in China [22].

Age	Cancers																		Survival
	A	B	C	D	E	F	G	H	I	J	K	L	M	N	O	P	Q	R	
0-	0	0	0	1	14	0	1	0	0	0	0	0	0	0	10	88	15	129	2804289
1-	0	0	0	0	32	0	2	1	0	0	0	0	0	0	42	239	31	347	11836631
5-	5	0	1	0	13	2	2	0	0	0	0	0	0	1	39	217	18	298	15322497
10-	4	1	2	2	12	1	5	2	0	0	0	2	0	0	42	207	11	291	12042350
15-	9	1	5	18	33	1	20	5	1	0	2	4	0	1	52	265	14	431	14609389
20-	19	5	33	39	121	10	48	5	9	8	4	8	1	3	57	327	21	718	22895079
25-	31	9	125	84	301	21	160	10	70	38	15	16	2	4	128	380	35	1429	18670345
30-	77	14	209	168	671	43	280	18	165	112	28	34	2	8	99	322	44	2294	15611678
35-	152	43	385	292	1405	72	660	17	335	187	74	53	4	13	143	352	59	4246	19793806
40-	338	245	953	623	3386	241	1957	62	780	465	128	136	10	34	288	508	119	10273	22967145
45-	582	792	1943	1104	5582	479	4174	78	1185	836	254	223	15	62	454	624	153	18540	26394480
50-	737	1573	2899	1470	6787	759	6897	79	1408	938	300	351	44	128	632	670	220	25892	16034245
55-	804	2613	4376	2035	7964	1231	10417	108	1343	800	341	331	86	217	774	730	250	34420	18285929
60-	1025	4531	7151	3098	9985	1703	16323	153	1360	892	329	416	211	397	1109	1065	337	50085	14053811
65-	900	5299	8031	3362	8808	1832	17509	192	861	716	339	349	281	520	1070	896	339	51304	9032555
70-	733	5371	8314	3536	7551	1826	17546	202	652	594	228	273	508	658	1041	860	309	50202	6728141
75-	694	5160	8422	4052	6600	1743	17766	237	587	518	229	211	840	891	932	815	385	50082	5350578
80-	507	4122	6673	3645	4893	1424	13547	298	469	367	182	147	887	887	645	528	382	39603	3320829
>85	329	2607	4366	2727	3064	869	8259	361	405	250	99	88	826	830	335	270	282	25967	1817600

Neoplasms (ICD-10 codes): A: lip, oral cavity, pharynx (C00–C14); B: esophagus (C15); C: stomach (C16); D: colon, rectum (C18–C21); E: liver (C22); F: pancreas (C25); G: trachea, lung (C33–C34); H: skin (C43–C44); I: breast (C50); J: cervix (C53); K: uterus (C54–C55); L: ovary (C56); M: prostate (C61); N: bladder (C67); O: lymphoid, hematopoietic, related tissue (C81–C90, C96); P: leukemia (C91–C95); Q: other malignant neoplasms (C17, C23–C24, C26–C32, C37–C41, C45–C49, C51–C52, C57–C60, C62–C66, C68–C80, and C97); R: all cancers.

**Table 3:** Two time point analysis of the mortality rate for different cancers.

Cancers	Standardized mortality rate (annual deaths per 10^5^ population)		RC	lnRC	*p*
	2004	2015			
Lip, oral cavity, and pharynx	2.07	1.87	0.90	−0.11	<0.001
Esophagus	14.02	8.21	0.59	−0.53	<0.001
Stomach	22.89	13.80	0.60	−0.51	<0.001
Colon and rectum	6.62	6.68	1.01	0.01	<0.001
Liver	24.28	18.01	0.74	−0.30	<0.001
Pancreas	2.37	3.14	1.32	0.28	<0.001
Trachea and lung	27.41	29.59	1.08	0.08	<0.001
Skin	0.61	0.45	0.74	−0.30	<0.001
Breast	2.57	2.64	1.03	0.03	<0.001
Cervix	1.26	1.85	1.47	0.39	<0.001
Uterus	1.95	0.69	0.35	−1.05	<0.001
Ovary	0.65	0.72	1.11	0.10	<0.001
Prostate	0.82	0.85	1.04	0.04	<0.001
Bladder	1.22	1.12	0.92	−0.08	<0.001
Lymphoid, hematopoietic tissue	1.52	2.20	1.45	0.37	<0.001
Leukemia	3.69	3.03	0.82	−0.20	<0.001
Other malignant neoplasm	1.59	0.81	0.51	−0.67	<0.001
All cancers	123.90	106.14	0.86	−0.15	<0.001

lnRC is the absolute RC (logarithmically transformed RC). A value <1 indicated a decreasing trend of mortality with time.

**Table 4:** The mortality risk from aging with time for major cancers using Poisson regression analysis.

Group of cancers		B	95% CI	p
Undeveloped	Uterus	−0.144	−0.272~−0.017	0.027
	Other malignancy	0.090	−0.046~0.225	0.196
	Esophagus	0.164	0.112~0.216	<0.0001
	Stomach	0.020	−0.019~0.059	0.322

Insensitive to social development	Skin	−0.085	−0.317~0.147	0.475
	Liver	−0.052	−0.085~−0.019	0.002
	Leukemia	0.435	0.343~0.528	<0.0001
	Lip, oral cavity, and pharynx	−0.040	−0.130~0.051	0.390
	Bladder	−0.038	−0.245~0.168	0.717
	Colon and rectum	0.009	−0.058~0.075	0.798
	Breast	−0.023	−0.123~0.076	0.647
	Prostate	0.282	−0.037~0.601	0.084
	Lung	−0.028	−0.061~0.005	0.093
	Ovary	−0.041	−0.232~0.149	0.670

Developed	Pancreas	−0.124	−0.233~−0.014	0.027
	Lymphoid, hematopoietic tissue	−0.005	−0.130~0.119	0.931
	Cervix	−0.169	−0.304~0.034	0.014
	All cancers	−0.002	−0.017~0.014	0.809

B: measure of the interaction between time and age (a positive value of B indicates an increased aging risk with time); CI: confidence interval.

## Data Availability

The data used to support the findings of this study are available from the corresponding author upon request.
